# Cardiac Capillary Hemangioma in a Patient Presenting with Syncope: Report of a Case and Systematic Review of the Literature

**DOI:** 10.3390/jcm15218372

**Published:** 2026-10-27

**Authors:** George Bahna, Abbas Agaimy, Robert Cesnjevar, Frank Harig, Ann-Sophie Kaemmerer-Suleiman, Sebastian Rauch, Timo Seitz, Oliver Dewald, Mathieu N. Suleiman

**Affiliations:** 1 Department of Cardiac Surgery, University Hospital Erlangen, Friedrich-Alexander-University Erlangen-Nürnberg, 91054 Erlangen, Germany; 2 Department of Pathology, Institute of Pathology, Erlangen University Hospital, Friedrich-Alexander-University Erlangen-Nürnberg, 91054 Erlangen, Germany; 3 Klinik für Herz- und Gefäßchirurgie am Klinikum Karlsburg, Herz- und Diabeteszentrum MV, 17495 Karlsburg, Germany

**Keywords:** primary cardiac tumor, hemangioma, cardiac surgery, benign cardiac tumors

## Abstract

**Background**: Primary cardiac tumors (PCTs) are exceptionally rare, accounting for less than 0.7% of all cardiac masses. Among these, cardiac hemangiomas are uncommon benign neoplasms, accounting for less than 2% of all PCTs, with the capillary subtype representing only a small fraction. Clinical presentation is often nonspecific and depends on the size, location, and mobility of the tumor. Syncope as the initial manifestation is exceedingly rare. **Case Summary**: We report the case of a 53-year-old woman who presented with recurrent episodes of syncope and no preceding cardiac symptoms. Initial neurological investigations were unremarkable. Transthoracic echocardiography revealed a mobile, pedunculated intraventricular mass attached to the mid-ventricular septum near the posteromedial papillary muscle. Cardiac MRI confirmed a well-defined, hypervascular lesion consistent with a benign cardiac tumor, initially suspected to be cardiac myxoma. The patient underwent successful surgical excision via superior partial sternotomy. Histopathological and immunohistochemical analyses confirmed the diagnosis of a capillary hemangioma. The postoperative course was uneventful, and postoperative imaging showed no recurrence. In addition, we provide a systematic review of the literature, identifying 46 studies comprising 49 patients with histopathologically confirmed cardiac capillary hemangiomas or mixed hemangiomas containing a capillary component. The review demonstrated considerable heterogeneity in patient age, clinical presentation, and tumor localization, with the right atrium and left ventricle being the most frequently affected sites. Surgical resection was the predominant treatment approach, while long-term follow-up data remained limited. **Discussion**: This case highlights the diagnostic challenge of distinguishing cardiac hemangiomas from other benign cardiac masses based solely on imaging. It underscores the importance of considering cardiac causes in patients presenting with unexplained syncope and the role of early echocardiographic evaluation. Definitive diagnosis requires histopathological confirmation, and surgical resection may be beneficial to prevent hemodynamic or embolic complications and to establish tissue diagnosis. Our systematic review also shows that diagnosing these rare tumors remains challenging, as their clinical presentations vary widely and their imaging features often resemble those of other cardiac masses.

## 1. Introduction

Primary cardiac tumors are exceedingly rare, with an estimated prevalence of 0.001–0.03% in autopsy series [1]. The vast majority are benign most commonly myxomas, followed by lipomas, papillary fibroelastomas, and hemangiomas [2,3].

Cardiac hemangiomas account for less than 2% of cardiac neoplasms and may arise from any cardiac chamber or layer, including the endocardium, myocardium, or pericardium [4].

Histologically, cardiac hemangiomas are classified as cavernous, capillary, or arteriovenous, while the arteriovenous subtype represent the rarest form. These lesions are typically well circumscribed and composed of proliferating endothelial cells forming capillary-sized vascular channels. While often being asymptomatic and incidentally detected, they still may cause clinical symptoms through obstruction of blood flow, interference with valvular function or conduction system, or embolization.

Syncope as the initial manifestation of a cardiac hemangioma is particularly uncommon and may easily be misattributed to neurological or vasovagal causes. Early cardiac imaging, especially transthoracic echocardiography, plays a pivotal role in the diagnostic work-up of unexplained syncope.

We present a rare case of a cardiac capillary hemangioma located in the left ventricle, initially suspected to be a myxoma, in a patient who presented with recurrent syncopal episodes. A systematic review of the literature on cardiac capillary hemangiomas is also provided.

## 2. Case Presentation

A 53-year-old woman (171 cm, 90 kg, BMI: 30.8 kg/m^2^) presented to the emergency department of a regional hospital after experiencing syncope at home. After initial spontaneous recovery within a few minutes, a second episode occurred a few hours later, for which the patient reported a prolonged period of unconsciousness of approximately two hours. Both episodes occurred while standing and without any prodrome. There were no accompanying symptoms such as dyspnea, chest pain, or palpitations. The admission ECG showed sinus rhythm without relevant abnormalities. Orthostatic measurements and family history were not documented.

The patient’s past medical history included closure of a patent foramen ovale (PFO) after an ischemic cerebral stroke, as well as chronic lumbosciatic pain syndrome secondary to lumbar disc herniation. Cardiovascular risk factors included active nicotine consumption and elevated lipoprotein(a) levels.

After initial evaluation in the emergency department, a cranial and cervical spine CT scan was performed, showing no evidence of acute trauma or other pathology. Neurological consultation suspected drop attacks due to posterior circulation hypoperfusion, possibly related to a subclavian steal syndrome. Given the inconclusive findings on initial CT imaging, a contrast-enhanced brain MRI and a duplex sonography of the supra-aortic arteries were obtained for further neurological assessment. These studies revealed no relevant cerebrovascular abnormalities besides old chronic lacunar defects being observed bilaterally in the cerebellum. A 24 h Holter ECG did not document any relevant cardiac arrhythmias.

**Figure 1:**
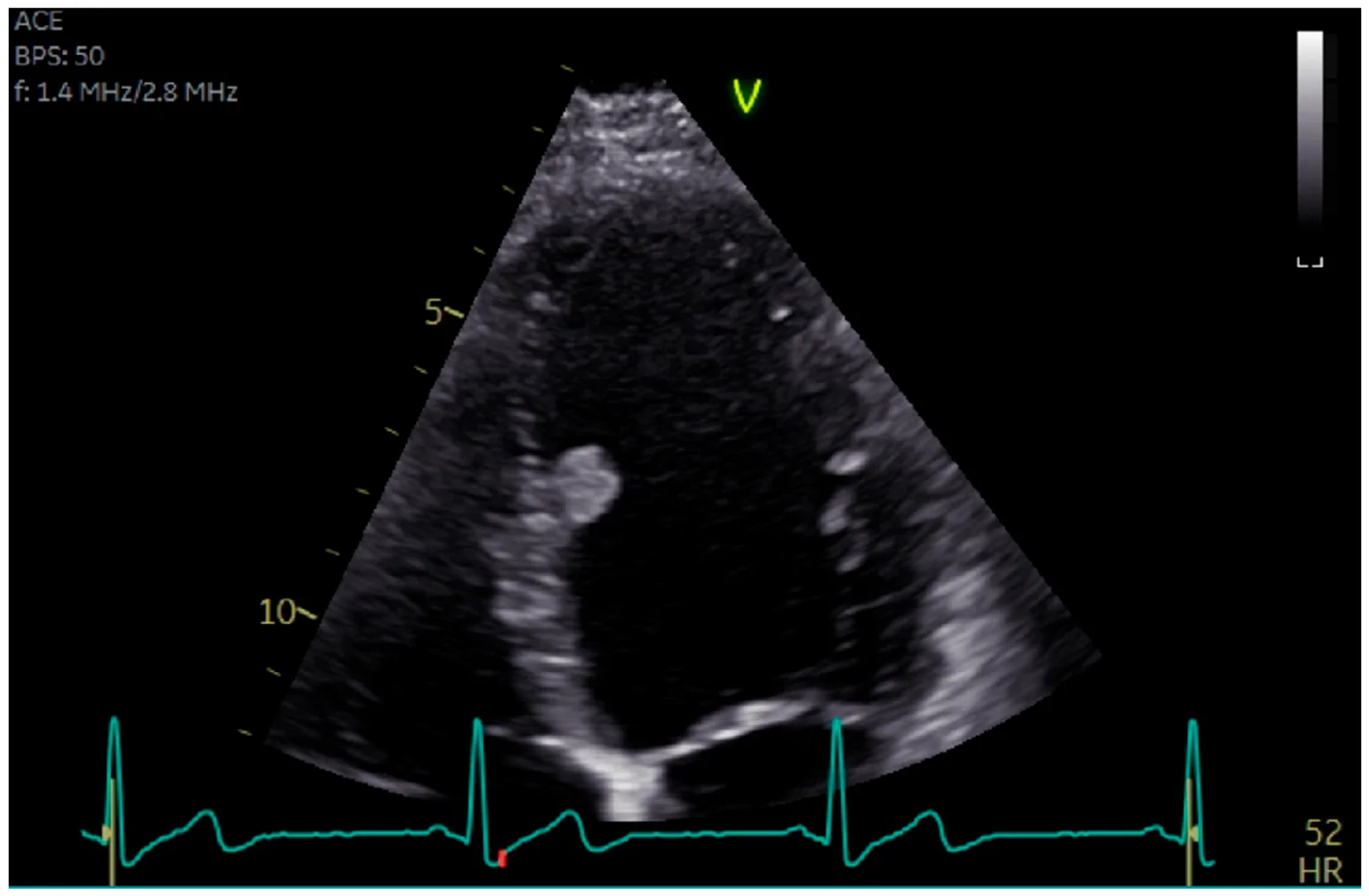
Transthoracic echocardiography (TTE), four-chamber view, showing a pedunculated 11 × 11 mm tumor in the left ventricle, attached to the septum.

A transthoracic echocardiography (TTE) ruled out pericardial effusion. The left ventricle was of normal size and geometry, with preserved systolic function. The left atrium was mildly dilated. However, 35 mm below the aortic valve, a pedunculated, 11 × 11 mm mobile mass was found attached to the middle of the left-sided interventricular septum and near the posteromedial papillary muscle. The mass caused no obstruction of the left-ventricular outflow tract at rest or during Valsalva maneuver (Figure 1).

**Figure 2:**
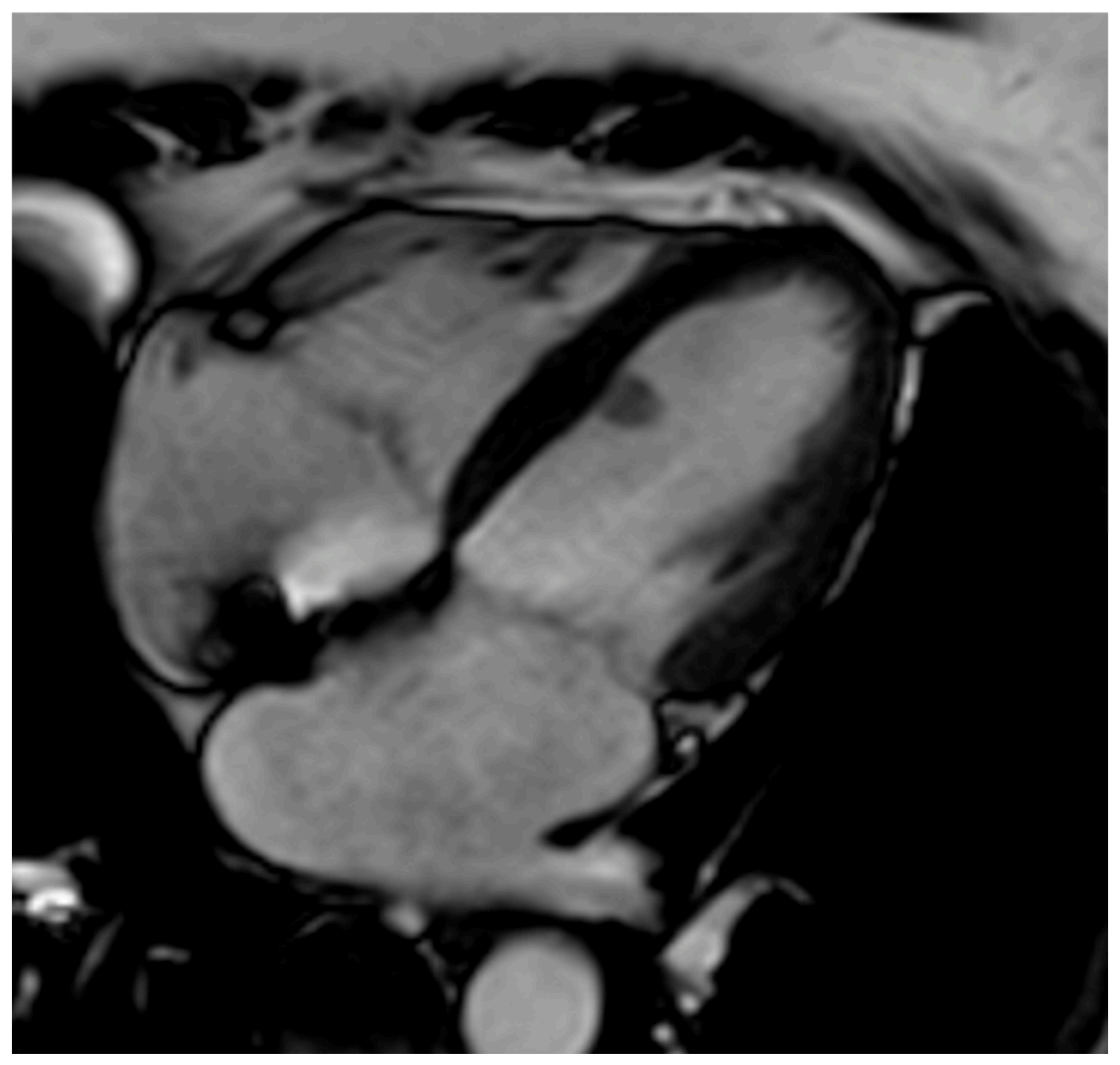
Cardiac MRI demonstrating the tumor measuring 11 × 9 mm.

Cardiac MRI revealed a normal left-ventricular size and function and a mild mitral regurgitation. At the mid-ventricular septum, cine imaging demonstrated a well-demarcated, benign-appearing, mobile mass of 11 × 9 mm. The lesion was iso-intense on T1-weighted images before contrast administration and appeared as hyper-intense on T2-weighted images, with strong perfusion and contrast enhancement (Figure 2). Late gadolinium enhancement of the myocardium showed no scarring or fibrosis. A preoperative transesophageal echocardiography (TEE) confirmed the previous diagnosis. Based on all imaging characteristics, the most likely diagnosis was a cardiac myxoma. The patient was referred to our tertiary cardiac surgery center for treatment. On admission, she denied any further syncopal episodes and was hemodynamically stable without signs of heart failure.

Due to the location and mobility of the intraventricular mass, operative treatment was indicated to prevent hemodynamic compromise or potential thromboembolic events. The patient underwent cardiac surgery through a superior partial sternotomy under normothermic cardiopulmonary bypass and cardioplegic arrest.

**Figure 3:**
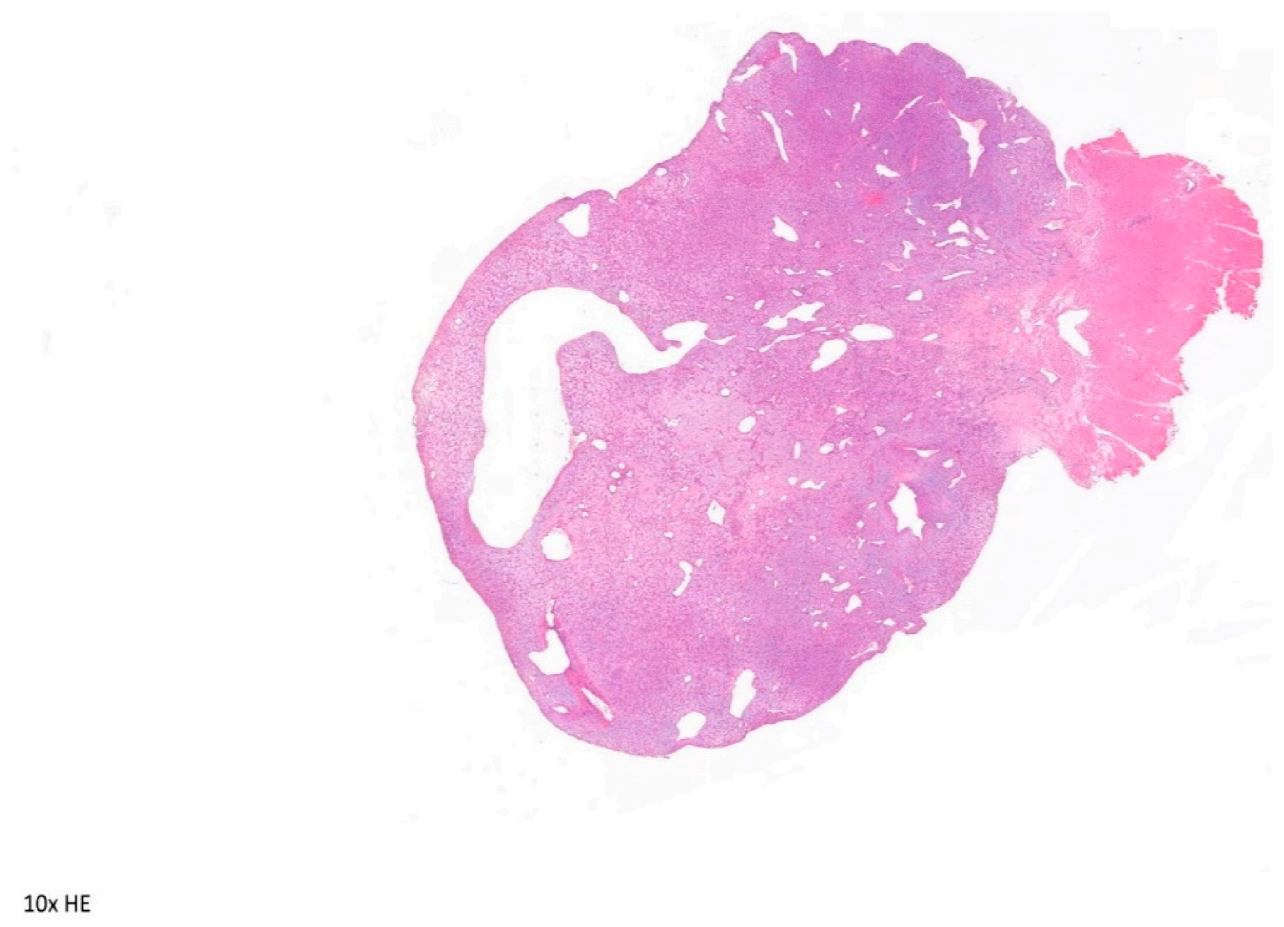
An overview of the polypoid, endoluminal mass showing its origin from the myocardium.

**Figure 4:**
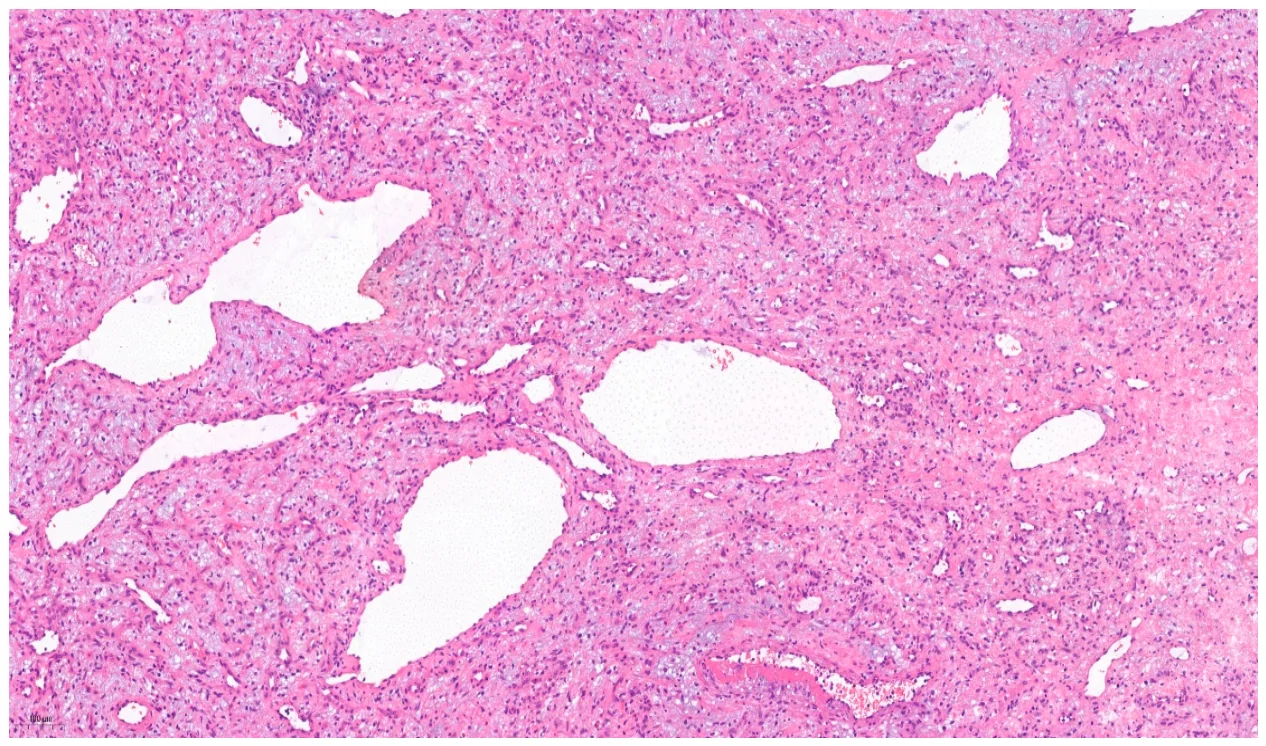
The lesion consists of capillary-type, vascular channels embedded in a loose fibromyxoid stroma with no evidence of cytological atypia.

**Figure 5:**
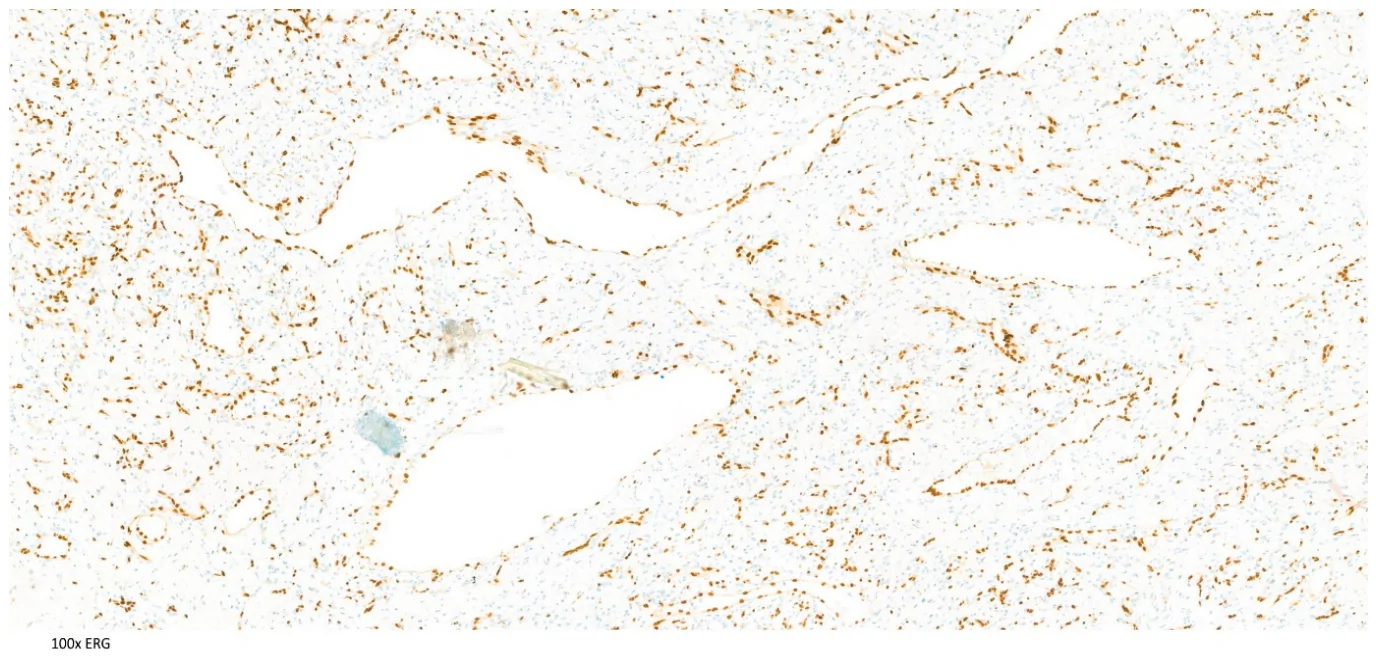
ERG showing strong nuclear positivity in the endothelial lining of the capillary vessels.

A transverse aortotomy was performed, and the tumor was accessed through the native tricuspid aortic valve. A mobile, pedunculated 12 mm measuring mass was visualized, originating from the left side of the mid-interventricular septum near the posteromedial papillary muscle. The tumor was excised in toto (Figure 3, Figure 4 and Figure 5). After thorough deairing and closure of the aortotomy, the patient was uneventfully weaned from cardiopulmonary bypass. Total aortic cross-clamp and cardiopulmonary bypass times were 17 and 36 min, respectively.

The postoperative course was uneventful. Postoperative transthoracic echocardiography showed no residual intraventricular mass, a normally dimensioned left ventricle with preserved systolic function, and no pericardial effusion.

Pathological examination showed a polypoid, endoluminal mass, covered by unremarkable endocardium. The lesion originated from the myocardium and consisted of capillary-type, vasoformative vascular proliferations embedded in a loose fibromyxoid stroma. The vascular proliferations appeared regular, without evidence of architectural or cytological atypia or necrotic areas. Peripherally, a larger central vessel could be identified from which the capillary structures arborized. The adjacent myocardium exhibited only minor variations in cardiomyocyte size, without showing evidence of inflammatory infiltrates or fibrotic remodeling.

The differential diagnosis of the lesion encompasses myxoma and angiosarcoma; both were excluded by defined morphological criteria.

## 3. Materials and Methods

### 3.1. Literature Search

**Figure 6:**
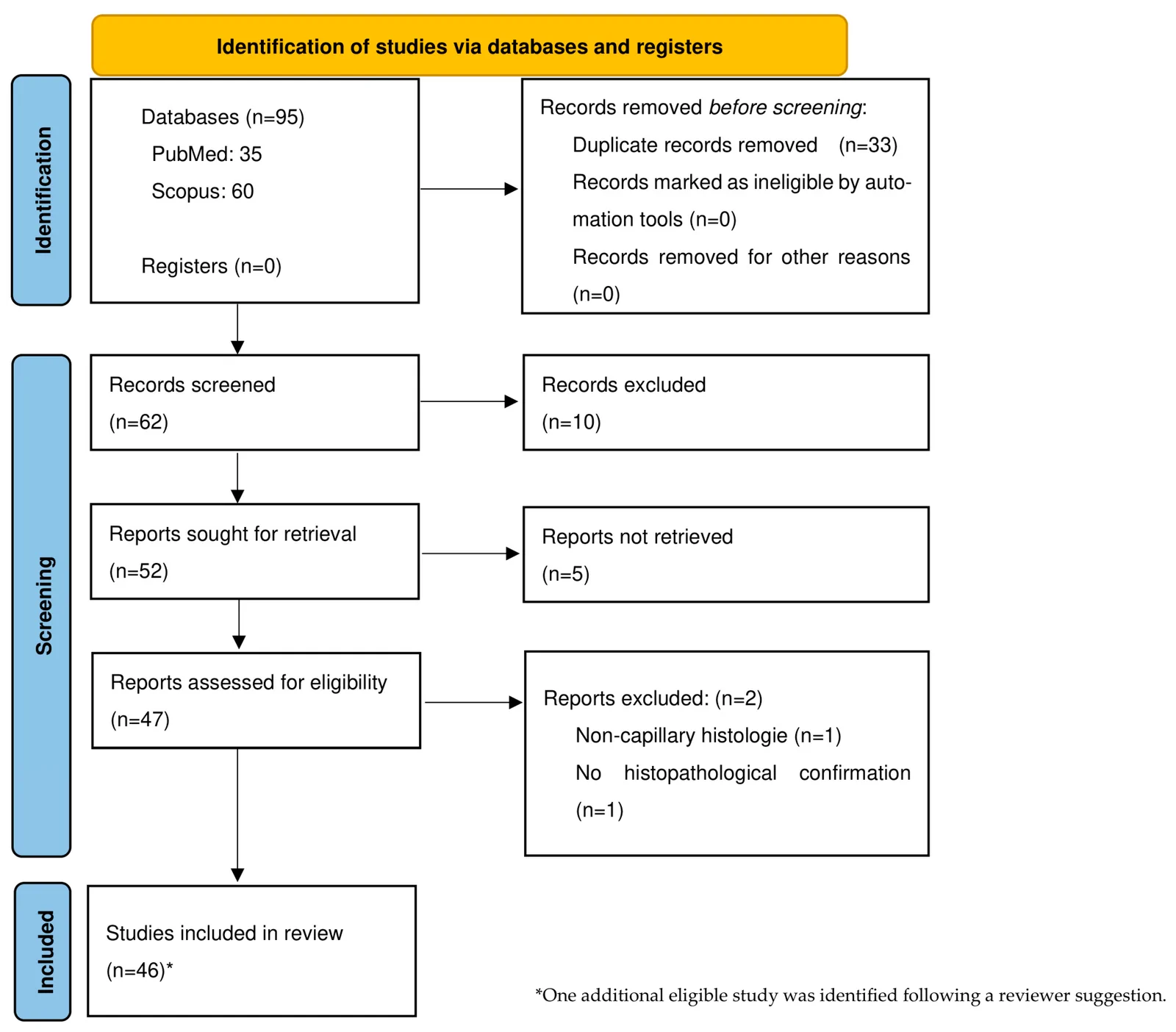
PRISMA 2020 flow diagram of the study selection process.

This systematic review was performed in accordance with the PRISMA 2020 checklist guidelines (Supplementary Material). The review protocol was not prospectively registered. The study selection process is illustrated in Figure 6. A systematic search of the PubMed and Scopus databases was conducted using the keywords “cardiac hemangioma” and “capillary”. All articles published in English or German up to 21 September 2026 were considered. Two authors (G.B. and M.S.) independently screened all identified records. Full texts of the potentially relevant studies were retrieved and reviewed in detail.

### 3.2. Eligibility Criteria and Data Extraction

Studies were considered eligible if they were published in English or German, involved the term “cardiac hemangioma”, and provided histopathological confirmation of the capillary subtype or a mixed subtype containing capillary components. Studies were excluded if they lacked histopathological confirmation, reported other types of cardiac tumors or involved hemangiomas of a subtype other than capillary.

Data extraction was performed by two authors (G.B and MS). The predefined variables extracted included patient age and sex, clinical presentation, tumor localization and size, diagnostic modality, surgical approach and cannulation strategy, histological subtype, and any additional relevant information. Uncertainties were discussed among the authors. The extracted data were summarized using counts and percentages where appropriate. Because the included reports were clinically heterogeneous, the findings were also summarized narratively by describing the main patterns across the reported cases. No formal methodological quality, reporting bias, certainty of evidence or risk-of-bias assessment was performed.

### 3.3. Study Identification and Selection

A total of 95 records were identified through database searches, including 35 from PubMed and 60 from Scopus. After removal of 33 duplicates, 62 unique records remained for title and abstract screening. At this stage, 10 records were excluded. Full texts were sought for the remaining 52 reports, of which 5 could not be retrieved. Therefore, 47 reports underwent full-text assessment. Two reports were subsequently excluded: one because the histological subtype was non-capillary and one because histopathological confirmation was lacking. No additional eligible articles were identified through reference list screening. Ultimately, 45 studies fulfilled the inclusion criteria through our screening process. One additional eligible study, which had not been identified by the original database search, was added following a peer-review recommendation, resulting in a total of 46 included studies.

## 4. Results

### 4.1. Patient Characteristics and Clinical Presentation

The 46 included studies comprised 49 individual patients. A total of 36 patients (73.5%) were adults, while 13 (26.5%) were younger than 18 years. Among adult patients, the mean age was 53.7 years (range, 21–78 years). Sex was reported for 39 patients, of whom 21 (53.8%) were male and 18 (46.2%) were female.

Clinical presentation was heterogeneous. Dyspnea was reported in 12 cases (24.5%), chest pain or chest discomfort in six (12.2%), and palpitations in five (10.2%). Recurrent pericardial effusion was described in four cases (8.2%), while cardiac murmurs were reported in three (6.1%). Syncope was reported in only one case (2.0%). Other notable presentations included cardiac arrest, supraventricular tachycardia, ventricular arrhythmias, and sudden cardiac death. Seven cases (14.3%) were detected prenatally.

Of particular note, three cases [5,6,7] were incidentally detected during follow-up for gastrointestinal malignancies (rectal, colon, and gastric cancer), and were all diagnosed as mixed cavernous–capillary hemangiomas. The demographic and clinical characteristics of the included patients are summarized in Table 1.

### 4.2. Anatomical Distribution and Diagnostic Imaging

The right atrium was the most frequently reported tumor location, accounting for 18 cases (36.7%), followed by the left ventricle in 16 cases (32.7%). Six tumors (12.2%) were located in the right ventricle or right ventricular outflow tract and two (4.1%) in the left atrium. The remaining cases involved other cardiac or paracardiac structures, including the mitral valve, pulmonary artery, and epicardium.

Among the 42 tumors assignable to a cardiac chamber, 24 (57.1%) were right-sided and 18 (42.9%) were left-sided. Ventricular involvement was reported in 22 cases (52.4%) and atrial involvement in 20 cases (47.6%).

In a published case report [7], the mass was not visualized on TTE but was detected on follow-up CT and subsequently confirmed by intraoperative TEE. Preoperative misclassification may also occur: in one case [8], a myxoma was suspected, in another case [9], the mass was misinterpreted as a cardiac cyst, while in a third case [7], the mass was treated as an intracardiac thrombus for six months without regression signs.

Transthoracic echocardiography was the most frequently reported diagnostic modality, followed by computed tomography and cardiac magnetic resonance imaging.

Many patients had more than one imaging modality during the diagnostic work-up.

### 4.3. Surgical Management and Outcomes

Surgical resection was commonly reported, with median sternotomy representing the most frequently described surgical approach. Minimally invasive techniques, including right minithoracotomy and robotic-assisted resection, were reported in individual cases.

Surgical strategies varied according to tumor location and extent. Notable cases included mitral valve replacement, reconstruction of the left main coronary artery and left ventricular wall, and intentional incomplete resection to avoid atrioventricular conduction injury.

Three deaths were documented among the reported cases. Reported postoperative complications included right heart failure, atrial fibrillation, and persistent electrocardiographic abnormalities. Long-term follow-up was inconsistently reported, preventing a reliable assessment of recurrence and long-term survival.

## 5. Summary of Included Studies

A detailed overview of the 46 included studies is provided in Table 1.

**Table 1:** Characteristics and findings.

Case	Sex/Age	Presentation	Localization/Size	Diagnostic Modality	Surgical Approach and Cannulation	**Histology**	**Notes**
Anbardar et al., 2022 [10]	Second case 58 female	Chest pain	Right atrium (2 × 1 × 1 cm)	TTE	Median sternotomy	Capillary hemangioma	CABG also performed
Darbari et al., 2020 [11]	48 female	Dyspnea, chest discomfort; recurrent pericardial effusion	Superior to left ventricle and posterolateral to main pulmonary artery infundibulum (43 × 30 mm)	CT	Median sternotomy	Capillary hemangiomas	Not seen in TTE
Delisle et al., 2019 [12]	6-days-old neonate	Consciousness alterations	Basal lateral wall of the left ventricle, size ND	TTE	ND	Capillary hemangioma	Mass was actively bleeding within the pericardium
Di Valentino et al., 2006 [5]	62 male	TTE to establish EF before chemotherapy for colon cancer	Anterior medial left ventricular wall (16 × 8 mm)	TTE	Transsternal, video-assisted, in cardio pulmonary bypass and cardioplegic arrest	Cavernous–capillary hemangioma	Rapid growing. Size 1 mm/mo
Fathala 2012 [9]	23 male	Dyspnea	Left-ventricular mass	TTE	ND	Capillary hemangioma	Appearance like a cardiac cyst
Fu et al., 2024—case 12 [13]	Case 12 5-month-old female	ND	Right atrium, (1.5 × 2 × 1 cm)	TTE, MRI	Median sternotomy, aortic and biscaval cannulation	Capillary hemangioma	ND
Fu et al., 2026 [14]	46 male	ND	Left ventricle (13 × 13 mm)	TTE	ND	Capillary hemangioma	ND
Galzerano et al., 2023 [15]	21 male	Palpitations, atypical chest pain, dyspnea	Left ventricle attached to the midseptum (3 × 2 cm)	TTE	ND	Capillary hemangioma	ND
Gourmelon et al., 2024 [16]	76 male	Dyspnea	Posterior right atrium (47 × 37 × 37 mm)	TTE, CT	Right anterior minithoracotomy, bicaval cannulation	Capillary hemangioma	post-op right heart failure, atrial fibrillation and pneumonia
Han et al., 2014 [17]	61 female	Palpitations + fatigue	Posterior inferior wall of left ventricle (13 × 10 × 8 mm)	TTE	ND	Capillary hemangioma	ND
Haponiuk et al., 2017 [18]	Routine prenatal ultrasound suggested an anomaly echo, a mass in the right atrium	ND	Right atrium (3 × 2.5 cm)	TTE	Median sternotomy, bicaval cannulation	Capillary hemangioma	ASD closed, pre-op intermittently occluded via tumor
Hong et al., 2014 [8]	74 male	Dyspnea and chest discomfort	Left atrium (1.98 × 1.54 cm)	TTE	Median sternotomy, conventional cannulation, LAD CABG also done	Capillary hemangioma	ND
Iida et al., 2013 [6]	73 male	Incidental finding during follow-up for lung metastatic tumor after resection of rectal cancer	Surface of right ventricle (2.8 × 2.2 × 2.3 cm)	TTE	Median sternotomy bicaval cannulation	Cavernous–capillary hemangioma	ND
Karunasumetta et al., 2019 [19]	4-month-old female	Sudden dyspnea during breastfeeding	Right atrium (3 × 3.5 cm), causing right-ventricular inflow obstruction	TTE	Median sternotomy, bicaval cannulation, right atriotomy	Capillary and cavernous	ASD as an incidental finding
Kassi et al., 2013 [20]	30 male	Cardiac arrest	Lateral wall of left ventricle, size ND	TTE, CMR	No surgery	Capillary hemangiomas	Percutaneous biopsy was unsuccessful, so an open biopsy was performed
Kojima et al., 2003 [21]	First case 28 male	General fatigue	Anterolateral wall of left ventricle (13 × 11 × 10 mm)	TTE	ND	Capillary hemangioma	ND
Kojima et al., 2003 [21]	Second case 60 female	Medical checkup	Right atrium (30 × 18 × 15 mm)	TTE	ND	Capillary and cavernous hemangioma	ND
Ku et al., 2026 [22]	72 female	Referred after abnormal echocardiographic finding	Right atrium (5.8 × 4.7 cm)	TTE	ND	Capillary hemangioma	ND
Lee et al., 1996 [23]	32 female	Dyspnea, ascites and recurrent pericardial effusion	Diffuse involvement of the epicardium and great vessels	TTE	Median sternotomy	Capillary hemangioma	Cavernous component in parts of pericardium. Died from massive hemorrhage
Lin et al., 2015 [24]	Premature neonate	Prenatal detection	Right atrium, size ND	Fetal echocardiography	Median sternotomy	Capillary hemangioma	Prenatally diagnosed
Liu et al., 2014 [25]	53 unclear	Check up	Inferior wall of left ventricle (7.4 × 9.9 mm)	TTE	Left atriotomy through mitral valve	Capillary hemangioma	Retrospective tumor blush
Liu et al., 2025 [26]	47 female	Incidental finding	Right atrium (2.7 × 2.0 cm)	TTE, MRI	Median sternotomy, aortic cannulation	Capillary hemangioma	ND
Malakan Rad et al., 2018 [27]	1-day-old neonate	TTE after birth	Right atrium, 3.2 cm in largest diameter	Auscultation TTE	ND	Epitheloid–capillary hemangioma	Died at 3 days post-op
Manasse et al., 1999 [28]	75 female	Systo-diastolic aortic murmur	Left ventricle (3 × 3 cm)	TTE	Median sternotomy, bi-caval cannulation transventricular apical approach	Capillary hemangioma	ND
Mazen et al., 2016 [29]	27 female	Dyspnea and palpitations	Left-ventricular mass (1.3 × 1.7 cm)	TTE	Posterior interatrial approach	Capillary and sinusoidal hemangioma	ND
Miyoshi et al., 2020 [30]	76 male	ECG abnormalities, negative T waves	Anterolateral left-ventricular free wall (47 × 50 mm)	TTE, TEE, CT	No surgery	Capillary and cavernous hemangioma.	An open-chest needle biopsy with a minimal incision thoracotomy was performed
Murthy et al., 2012 [31]	53 male	“Abnormal” ECG during routine check up	Lateral wall of left ventricle, diameter 1.5 cm	TTE	Transatrial	Cavernous–capillary hemangioma	Supply from LAD and RCX tumor blush
Navas-blanco et al., 2018 [32]	76 male	Dyspnea	Distal aspect of the main pulmonary artery size ND	Coronary angiography, TTE	ND	Capillary hemangioma	ND
Qin et al., 2019 [33]	51 female	Chest pain	Right-ventricular outflow tract (2 × 2 × 1.8 cm)	TTE, CMR	ND	Capillary hemangioma	
Ranjan et al., 2023 [34]	52 male	Dyspnea	Right atrium 16 × 11 mm attached to the tricuspid-valve	TTE, TEE	Median sternotomy	Capillary hemangioma	ND
Reiss et al., 1991 [35]	28 female	Murmur during routine examinations	Right-ventricular outflow tract (3 × 3 cm)	TTE, MRI	Median sternotomy	Capillary–cavernous hemangioma	Nearly complete RV outflow tract obstruction
Rocco et al., 2024 [36]	78 male	Incidental finding	Anterior mitral valve leaflet (0.5 × 0.6 cm)	TTE, TEE	Right lateral thoracotomy, right femoral vein and right internal jugular vein cannulation. Arterial cannulation in the right femoral artery	Capillary hemangioma	Robotic minimally invasive approach
Sakai et al., 2016 [37]	40 female	Palpitations	Between antero-lateral und postero-medial papillary muscles (12.5 × 10.7 mm) attached to the inferior wall of the left ventricle	TTE	6 cm right anterolateral minithoracotomy; left atriotomy via the interatrial groove; femoral arterial cannulation; venous drainage via the SVC through the percutaneously cannulated right internal jugular vein and via the right common femoral vein	Capillary–cavernous hemangioma	ND
Sebastian et al., 2005 [38]	Prenatal ultrasound in the 25th week, pericardial effusion	ND	Free wall of the right atrium 12 × 13 to 19.5 × 18.8 mm	Fetal echo	At 24 h of life, median sternotomy, bicaval cannulation, right atriotomy, small secondary mass superior medial to the orifice of the coronary sinus not exceeded completely to avoid AV block	Capillary–cavernous	Echo 34 weeks of pregnancy, 23.5 × 24.9 mm rapid growth. In June 2025, unchanged residual from the tumor near the coronary sinus
Seifert et al., 2019 [39]	50 male	Recurrent pericardial effusion	Left atrium	MRI, CT	ND	Capillary hemangioma	ND
Seitz et al., 2018 [40]	48 female	Progressive dyspnea, recurrent pericardial effusion	Anterior wall of the left ventricle (20 × 22 × 8 mm)	TTE, CT	Anterolateral thoracotomy	Capillary hemangioma	ND
Seo et al., 2023 [41]	69 male	CT for elevated liver enzyme	Right atrium, size ND	CT, TEE	ND	Capillary hemangioma	ND
Sharma et al., 2009—case 1 [42]	Prenatal ultrasound in the 32nd week, large mass	Prenatal detection	Right atrium, size ND	Fetal echocardiography	On the 5th day of life through a median sternotomy without cardiopulmonary bypass	Capillary hemangioma	Vascular clamp from outside without cardiopulmonary bypass
Sharma et al., 2009—case 2 [42]	Prenatally diagnosed in the 33rd week	Prenatal detection, worsening fetal cardiac failure	Right atrium, size ND	Fetal echocardiography	On the 3rd day of life through a median sternotomy, ascending aortic and right atrial cannulation	Capillary hemangioma	Elective caesarean section was then performed at 34 weeks because of worsening fetal cardiac failure
Sharma et al., 2009—case 3 [42]	Prenatally diagnosed in the 20th week atrial mass and significant pericardial effusion.	Prenatal detection	Right atrium, size ND	Fetal echocardiography	On the 5th day of life through a median sternotomy. Cardiopulmonary bypass using aortic cannulation, and a single venous cannula being inserted at the junction between the inferior caval vein and the right atrium	Capillary hemangioma	Caesarean section at 35 weeks because of exaggerated reversal of flow with atrial systole in the inferior caval vein, with an increase in the size of the tumor.
Sogawa et al., 2012 [7]	73 male	CT follow-up after gastric cancer diagnosis	Right-ventricular outflow tract (RVOT) just below the pulmonary valve	CT	Median sternotomy, bicaval cannulation, incision of the main pulmonary artery	Cavernous–capillary hemangioma	Possibility that the mass is thrombus, 6 months of warfarin, follow-up CT no change, TTE did not show the tumor, intraoperative TEE did
Spanò et al., 2015 [43]	47 male	During the endovascular procedure development of SVT	Right atrium (9 × 7.5 cm)	TTE	ND	Cavernous capillary	No symptoms, although a huge mass occupied the whole right atrium
Takahashi et al., 2026 [44]	73 female	Systolic murmur	Right-ventricular free wall (44 × 24 × 24 mm)	TTE	ND	Capillary hemangioma	ND
Tomasian et al., 2007 [45]	16 female	Recurrent syncope	Right-ventricular free wall (11 × 10 mm)	TTE, CT	Median sternotomy	Capillary hemangioma	ND
Val-Bernal et al., 2017 [46]	62 male	Edema of lower limbs, His bundle block and T wave abnormalities in ECG	Anterior mitral valve leaflet (1.6 × 1.1 × 1.0 cm)	TTE	ND, mitral valve repair not possible; mitral valve replacement performed	Lymphocyte-rich capillary–cavernous hemangioma	ECG abnormalities persisting postoperative
Wang et al., 2017 [47]	42 male	Palpitations	Lateral wall of left ventricle (15 × 10 × 10 mm)	TTE	ND	Capillary hemangioma	Pre-op bigeminus and PVCs post-op SR, no bigeminus
Yildirim et al., 2021 [48]	49 female	Dyspnea and chest pain	Left paracardiac mass extending from the pulmonary conus to the heart base, involving regions of the left atrium and ventricle without invading the heart (90 × 65 mm)	CT, TTE, CMR	Median sternotomy, aortic and two-stage unicaval cannulation	Capillary hemangioma	Complete excision required resection of a 4 cm LMCA segment; LMCA reconstructed with an interposed saphenous vein graft and LV wall repaired with an autologous pericardial patch
Zerbo et al., 2009 [49]	15 male	Sudden death	Intramural 3 cm located at the apex	autopsy	No surgery	Capillary and arteriolar	ND
Zhou et al. 2026 —case 1 [50]	Prenatally diagnosed in the 37 + 5th week	Prenatal detection	Right atrium (13 × 17 × 24 mm)	Fetal echocardiography	Median sternotomy	Capillary hemangioma	ND

Abbreviations: ASD, atrial septal defect; AV, atrioventricular; CABG, coronary artery bypass grafting; CMR, cardiac magnetic resonance; CT, computed tomography; ECG, electrocardiogram; EF, ejection fraction; LAD, left anterior descending coronary artery; LMCA, left main coronary artery; LV, left ventricle; MRI, magnetic resonance imaging; ND, not disclosed; PVC, premature ventricular contraction; RCX, circumflex coronary artery; RVOT, right-ventricular outflow tract; SR, sinus rhythm; SVC, superior vena cava; SVT, supraventricular tachycardia; TEE, transesophageal echocardiography; TTE, transthoracic echocardiography. Studies are listed alphabetically according to the surname of the first author.

## 6. Discussion

This case report aims to highlight the exceptional rarity of cardiac hemangioma, especially the capillary subtype, and to emphasize that preoperative diagnostic suspicion does not always match the final histological findings. It also underscores the need for an early evaluation of potential cardiac causes in patients with non-specific symptoms.

We report the exceptional case of a cardiac hemangioma located in the middle of the left ventricle, initially suspected to be a myxoma. Although the cardiac mass was identified during the diagnostic work-up for recurrent syncope, a direct causal relationship between the tumor and the syncopal episodes could not be established.

**Figure 7:**
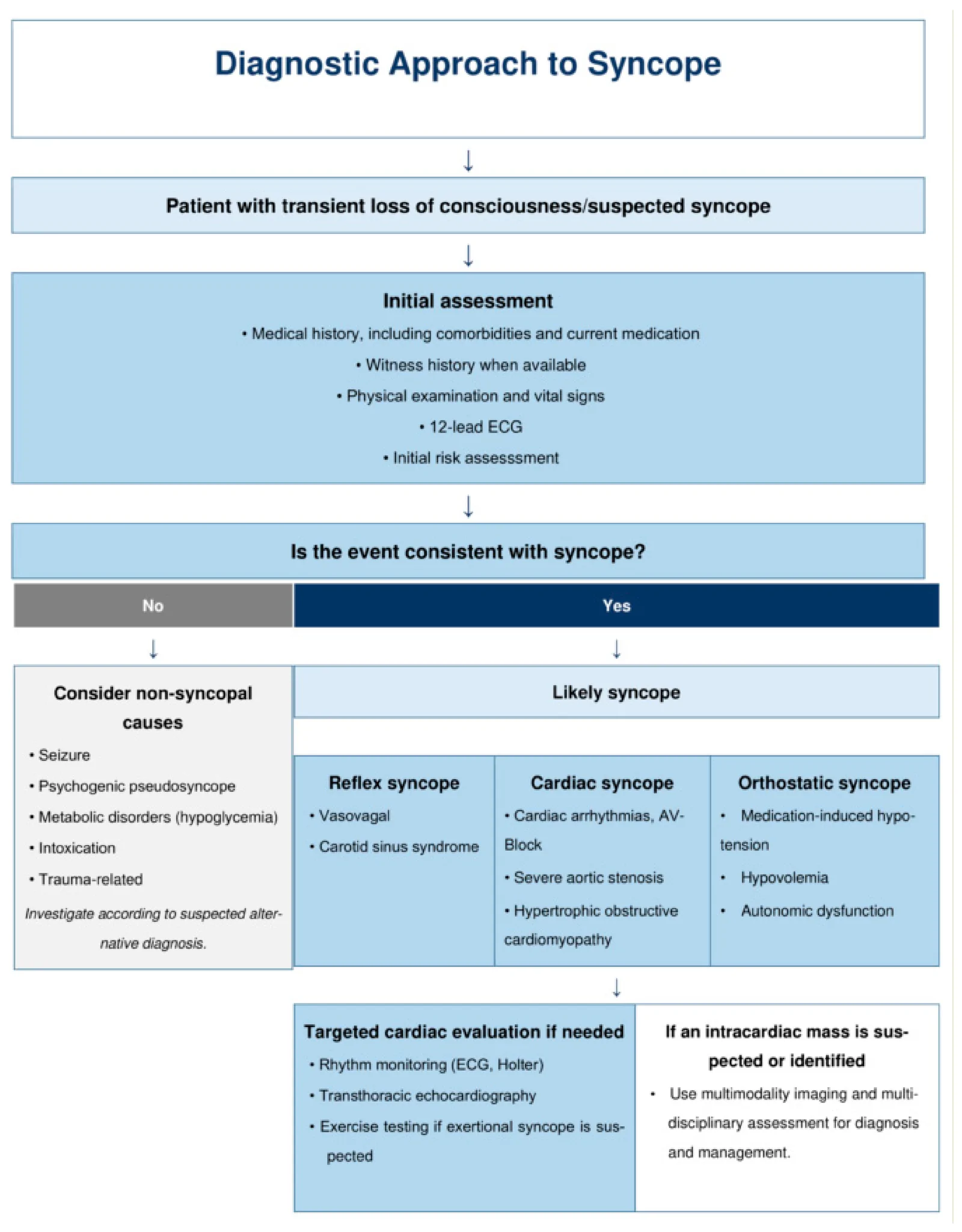
Simplified diagnostic approach to syncope, not representing the full guideline algorithm. Sources: [54,55,56].

Syncope has a broad differential diagnosis. Common forms of reflex syncope include vasovagal, situational, and carotid sinus syncope [51]. Orthostatic syncope can result from medication-induced hypotension, autonomic failure, or hypovolemia [52]. Cardiac arrhythmias are another important cause of syncope and may coexist with structural heart disease [53]. Therefore, a 24 h Holter ECG was performed in our patient to exclude relevant arrhythmias and atrioventricular block. Several conditions may also mimic syncope, including hypoglycemia, psychogenic pseudosyncope, and cerebrovascular events such as transient ischemic attack. A structured diagnostic work-up is therefore important to identify the underlying cause and determine which patients require further cardiac evaluation, as outlined in Figure 7.

In this case, the well-defined margins, stalk-like attachment, and the absence of infiltrative features on preoperative echocardiography suggested a benign lesion. However, given the clinical symptoms and the unusual location of the tumor in the left-ventricular cavity, surgical removal with subsequent histopathological analysis to exclude malignancy were indicated.

Cardiac hemangiomas are rare, representing less than 2% of all primary cardiac tumors [4]. These benign neoplasms arise from endothelial cells and show abnormal vascularization and increased blood supply. 

Histologically, hemangiomas are classified into capillary, cavernous, or arteriovenous types. The cavernous subtype, consisting of large blood-filled vascular spaces is the most frequent subtype (58%), while capillary hemangiomas account for only 9% of cardiac hemangiomas [57], being composed of numerous small vessels.

In general, clinical presentation of cardiac tumors, including hemangiomas, varies widely, depending on the tumor’s location, size, mobility, and histological entity. There is the potential to interfere with intracardiac blood flow or conduction system.

Symptoms may include dyspnea, chest pain, palpitations, embolic events, or syncope. Benign cardiac tumors frequently remain asymptomatic over extended period of time and are usually detected incidentally during routine imaging or work-up [58]. However, symptoms may arise from the tumor itself taking up space and causing congestion of surrounding tissue and structures, thereby interfering with normal cardiac function.

Primary malignant cardiac tumors, with sarcomas being the most common type, are exceedingly rare [59]. They may manifest with a range of symptoms, including heart failure, arrhythmias, and chest pain. Rapid progression is often accompanied by systemic manifestations or metastases at the time of diagnosis [60], while intracardiac thrombi, vegetations, or structural variants can mimic tumors and should therefore always be considered in the differential diagnosis.

The diagnosis of intracardiac masses relies primarily on imaging techniques such as transthoracic and transesophageal echocardiography. Cardiac MRI, CT or angiography provides additional information on size, location, tissue characteristics and mobility of the structure.

CMR differentiation of cardiac masses requires a multiparametric assessment of morphology, mobility, perfusion, and contrast-enhancement patterns. Unlike our lesion, thrombi are avascular and show no perfusion or enhancement. In our patient, T1 isointensity and T2 hyperintensity were compatible with the initial diagnosis of myxoma. However, myxomas typically show only mild perfusion [61], whereas our lesion demonstrated marked perfusion. The atypical mid-septal location further raised the possibility of an alternative diagnosis. The non-valvular location was also atypical for fibroelastoma and the absence of infiltrative features argued against a malignant tumor; the diagnosis was ultimately established by histopathology.

These observations highlight the non-specific imaging characteristics of hemangiomas and their overlap with other cardiac masses. This underlines the importance of histopathological examination for establishing a definitive diagnosis.

For symptomatic or mobile tumors, surgical resection is generally recommended, particularly when there is a risk for obstruction or thromboembolic complications. However, the decision for surgery should be individualized. Surgery could be also indicated to establish a definitive diagnosis. Alternatively, biopsy may be considered.

Despite the standard operative risks and the cardiac-surgery-specific risks associated with cardiopulmonary bypass and cardioplegia, surgical therapy still serves both diagnostic and therapeutic purposes.

## 7. Conclusions

The presented case highlights that the presumptive echocardiography- and MRI-based diagnosis of a benign cardiac tumor, often being a myxoma, can be misleading, especially when the tumor is located at an uncommon site such as the left ventricle. Furthermore, it demonstrates that intracardiac masses should be considered early into differential workup of unexplained syncope, especially when neurological and trauma-related investigations are non-revealing.

As the patient initially presented with recurrent episodes of syncope, the initial diagnostic approach focused on neurological and trauma-related causes. Since cranial and cervical CT scans, MRI angiography, and duplex sonography of the supra-aortic vessels provided no diagnosis, the subsequent cardiology work-up finally diagnosed a cardiac tumor.

Routine clinical and laboratory findings may provide non-specific clues, and therefore imaging plays the central role in diagnosis of cardiac tumors. Transthoracic echocardiography should be considered as an early, non-invasive diagnostic tool when a cardiac etiology is clinically suspected in patients with unexplained syncope. Transesophageal echocardiography, as well as cardiac MRI, offer additional detailed anatomical and functional insights. In selected cases, PET-CT may be valuable to detect potential malignancy and rule out metastatic disease. However, histopathology is essential to prove the dignity of the mass and provide definitive diagnosis and grading.

Therefore, surgical excision of resectable masses should be considered in the presence of mobility or obstruction or when associated with embolic or hemodynamic risk.

After successful treatment, regular follow-up imaging remains essential to monitor the outcome and possible tumor recurrence.

## Data Availability

All data supporting the findings of this study are included in the article.

## Supplementary Materials

The following supporting information can be downloaded at: https://www.mdpi.com/article/10.3390/jcm15218372/s1.
